# Cardioprotective and Metabolomic Profiling of Selected Medicinal Plants against Oxidative Stress

**DOI:** 10.1155/2018/9819360

**Published:** 2018-01-14

**Authors:** Nadia Afsheen, Khalil-ur-Rehman, Nazish Jahan, Misbah Ijaz, Asad Manzoor, Khalid Mahmood Khan, Saman Hina

**Affiliations:** ^1^Department of Biochemistry, University of Agriculture, Faisalabad, Pakistan; ^2^Department of Chemistry, University of Agriculture, Faisalabad, Pakistan; ^3^Department of Clinical Medicine and Surgery, University of Agriculture, Faisalabad, Pakistan

## Abstract

In this research work, the antioxidant and metabolomic profiling of seven selected medicinally important herbs including *Rauvolfia serpentina*, *Terminalia arjuna*, *Coriandrum sativum*, *Elettaria cardamom*, *Piper nigrum*, *Allium sativum*, and *Crataegus oxyacantha* was performed. The *in vivo* cardioprotective potential of these medicinal plants was evaluated against surgically induced oxidative stress through left anterior descending coronary artery ligation (LADCA) in dogs. The antioxidant profiling of these plants was done through DPPH and DNA protection assay. The *C. oxyacantha* and *T. arjuna* showed maximum antioxidant potential, while the *E. cardamom* showed poor antioxidative strength even at its high concentration. Different concentrations of extracts of the said plants exhibited the protection of plasmid DNA against H_2_O_2_ damage as compared to the plasmid DNA merely treated with H_2_O_2._ The metabolomic profiling through LC-MS analysis of these antioxidants revealed the presence of active secondary metabolites responsible for their antioxidant potential. During *in vivo* analysis, blood samples of all treatment groups were drawn at different time intervals to analyze the cardiac and hemodynamic parameters. The results depicted that the group pretreated with HC4 significantly sustained the level of CK-MB, SGOT, and LDH as well as hemodynamic parameters near to normal. The histopathological examination also confirmed the cardioprotective potential of HC4. Thus, the HC4 being safe and inexpensive cardioprotective herbal combination could be considered as an alternate of synthetic drugs.

## 1. Introduction

Oxidation is a natural phenomenon that leads to the formation of free radicals known as reactive oxygen species (ROS) [1]. Some of the ROS are very important in cell metabolism including intercellular signaling, phagocytosis, and energy production [2]. However, overproduction of ROS during biological processes resulted in extensive pathological alterations like DNA damage and various degenerative disorders. Humans are constantly exposed to natural DNA-damaging agents such as UV light, dietary agents, and endogenously formed free radicals. Damaged DNA accumulates in the brain, muscle, liver, kidney, and in long-lived stem cell, which causes aging, decline in gene expression, and loss of functional capacity [3].

Antioxidants are compounds that slow down or delay the oxidation process by obstructing the initiation of a series of oxidizing reactions [4]. Owing to the presence of antioxidants, medicinal plants have a shielding effect against various diseases, thus emerging as substantial therapeutic agents. Medicinal plants are a time-honored medicine used since the ancient era for treatment of various ailments in human beings [5]. Herbal medicines, in addition to their traditional values, also hold great public and medical interest worldwide as sources of novel lead compounds for drug development. Hence, the medicinal plants will be natural protective strategy and would be freely available with low cost as compared to synthetic drugs [6].

Pakistan is bestowed with a wide range of plant species with unique biodiversity in different climatic zones [7]. These medicinal plants have been used in scientific research for various cardiovascular disorder in human beings [8, 9]. Currently available synthetic cardioprotective drugs exhibit a number of side effects and are out of reach for poor communities. Cardioprotective effects of some medicinal plants, which are safe and inexpensive, have already been explored [10–13]. Therefore, the green products having cardioprotective and antioxidative potential have attracted many researchers towards metabolomic profiling and phytotherapy. The antioxidative strength of medicinal plants is because of the secondary metabolites present in it [11].

An LC-MS-based metabolomic study has become a powerful analytical tool for assessment of various secondary metabolites in herbal medicine [14]. These secondary metabolites have been found to possess a broad range of therapeutic properties, including antioxidant, cardioprotective, and antihypertensive potential [15]. A thorough integration of information from metabolomics is expected to provide solid evidence-based scientific rationales for the development of modern phytomedicines [16]. Therefore, in this research, the antioxidant potential, metabolomic profiling, and *in vivo* cardioprotective evaluation of *Rauvolfia serpentina*, *Terminalia arjuna*, *Coriandrum sativum*, *Elettaria cardamom*, *Piper nigrum*, *Allium sativum*, and *Crataegus oxyacantha* was done to get the potent role of these natural antioxidants in health. All these medicinal plants were selected as these plants have already been reported to possess cardiotonic, antioxidant, and antilipidemic potential [4, 17]. Moreover, the previous literature and the knowledge of CAM practitioners also endorsed the cardioprotective effect of these selected parts of the plants.

## 2. Materials and Methods

### 2.1. Preparation of Herbal Extract

Different parts of the medicinal plant like the roots of *R. serpentina*, bark of *T. arjuna*, seeds of *C. sativum* and *E. cardamom*, leaves of *P. nigrum*, and fruit of *A. sativum*, and *C. oxyacantha* were collected from the Botanical Garden of the University of Agriculture, Faisalabad, Pakistan and from the local herbal market. All the selected parts of the plants were identified by the plant taxonomist in the Department of Botany, University of Agriculture, Faisalabad, Pakistan. These parts of the plants were washed and pulverized to get fine powder. The powdered plants (5 g of each) were macerated in methanol (50 mL). The macerate was kept in an orbital shaker for four days. The supernatant was decanted and the residue was remacerated with methanol. The pooled supernatants were combined and filtered through Whatman's filter paper number 1. The rotary evaporator was used to concentrate the filtrate, and subsequently the filtrate was lyophilized [17].

### 2.2. Antioxidant Assay

#### 2.2.1. 1,1-Diphenyl-2-picrylhydrazyl (DPPH) Free Radical Scavenging Assay

The antioxidant potential was determined by using 1,1-diphenyl-2-picrylhydrazyl as a free radical. The methanolic solution of DPPH (0.1 mM) and plant extract of different concentrations (20, 40, 60, 80, and 100 *μ*g/mL) were mixed in equal volume. The mixtures was left for 30 minutes in the dark, and the absorbance was noted at 517 nm. Ascorbic acid was used as a standard. The percentage DPPH inhibition of plant extract was calculated as follows:

(1) $$\begin{array}{c} DPPH\;inhibition\left(\%\right)=\left[\frac{1-A_{1}}{A_{0}}\right]\times 100, \end{array}$$

where *A*_1_ is the absorbance of the sample, and *A*_0_ is the absorbance of control [4, 18].

#### 2.2.2. DNA Protection Assay

The DNA protection assay of extracts of different concentrations (100, 500, and 1000 *μ*g/mL) of selected plants was performed by using the pBR322 plasmid DNA. pBR 322 DNA plasmid (0.5 *μ*L) was diluted by using 50 mM sodium phosphate buffer (pH 7.4). After dilution, pBR 322 DNA (3 *μ*L) was treated with 5 *μ*L of selected concentrations of plant extracts. 4 *μ*L of 30% H_2_O_2_ was added to it, and sodium phosphate buffer (pH 7.4) was used to make the volume up to 15 *μ*L. The difference in migration between the oxidized and native DNA was observed on 1% agarose by horizontal DNA gel electrophoresis using a wide mini system (Techview, Singapore). 1% agarose was prepared by dissolving 1 g agarose in 100 mL of 1X × TAE buffer and placed in a microwave oven for two minutes. It was cooled and poured in a casting plate. After solidification, the gel was kept in the sodium phosphate buffer and samples were loaded in the wells one by one. The gel was stained with ethidium bromide and documented by Gene NuGenius unit Syngene model (Cambridge, UK). DNA protection was observed from DNA band corresponding to that of native in the presence and absence of various concentrations (100, 500, and 1000 *μ*L) of each plant's extract [19].

### 2.3. Metabolomic Profiling

Metabolomic profiling of all the selected medicinal plants was performed through liquid chromatography-mass spectrometry (LC-MS) analysis.

### 2.4. Liquid Chromatography-Mass Spectrometry (LC-MS)

The selected medicinal plants were analyzed by using liquid chromatography combined with electrospray ionization mass spectrometry (LC-ESI-MS). The plant extracts were filtered through a 0.45 *μ*m syringe filter before analysis. Separation was performed on Surveyor plus HPLC System equipped with Surveyor auto (Thermo Scientific, San Jose, CA, USA). The pump was equipped with a Luna Reverse Phase C-18 analytical column (4.6 × 150 mm, 3.0 *μ*m particle size) (Phenomenex, USA). Solvent elution consisted of LC-MS grade methanol and acidified water (0.5% formic acid *v*/*v*) as the mobile phase A and B, respectively. Solvent elution consisted of gradient system which runs at a flow rate of 0.3 mL/min. The gradient elution was programmed as follows: from 5 min in 15% A to 20 min in 25% B and maintained it till the end of the analysis. A 20 minute re-equilibration time was used after each analysis. The column was maintained at 25°C and the injection volume was 5.0 *μ*L. The effluent from the HPLC column was directed to an electrospray ionization mass spectrometer (LTQ XL™ linear ion trap, Thermo Scientific, River Oaks Parkway, USA). Parameters for analysis were set using negative ion mode with spectra acquired over a mass range from 260 to 2000 m/z. The optimum values of the ESI-MS parameters were spray voltage +4.0 kV, sheath gas and auxiliary gas were 45, and 5 units/min, respectively, capillary temperature 320°C, capillary voltage −20 V, and tube lens −66.51 V.s The accurate mass spectra data of the molecular ions was processed through the Xcalibur software (Thermo Fisher Scientific Inc, Waltham, MA, USA) [20].

### 2.5. Selection of Animals

The eighteen male stray dogs of 1-2 years were selected as experimental animals and acclimatized for one week under laboratory conditions (27°C in 12 hr dark/light cycle). All the animals were housed in the Animal House, Department of Clinical Medicine and Surgery, University of Agriculture, Faisalabad. They were fed with standard feed. The experiment was performed by taking the approval from the institutional ethics review board in the presence of a licensed veterinarian.

### 2.6. Herbal Combination Therapy

The four different herbal combinations of selected plant extracts were formed as given in Table 1. These herbal combinations were evaluated for their synergistic cardioprotective potential. The dogs were divided into three groups. The first group of dogs was the control group, to which normal diet was fed for 23 days. The second group was the positive control group, in which the dogs were treated with normal diet for 22 days, and after that, the ligation of the left anterior descending coronary artery (LADCA) was performed on the 23rd day. The third group was the treatment group which was further divided into four subgroups. Each subgroup was pretreated with its respective herbal combination (Table 1) for 22 days. On day 23, all the dogs of the treatment group underwent LADCA ligation. After completion of the surgical procedure, the blood samples were taken at various time intervals (0 to 48 hr) to analyze the cardiac markers (CK-MB, SGOT, and LDH). At the end of the experiment, the dogs were anesthetized by Sodium Pentothal and the hearts were excised for histopathological studies.

### 2.7. Surgical Induction of Myocardial Infarction

The dogs were anesthetized with Sodium Pentothal (20 mg/kg). Atropine was administered subcutaneously at a dose of 0.1 mg/kg once before the surgery to keep the heart rate elevated during the surgical procedure and to reduce the bronchotracheal secretions. The body temperature was monitored and maintained at 37°C. The animals were ventilated with room air from a positive pressure by using compressed air at the rate of 90 stroke/min and tidal volume of 10 mL/kg. The left jugular vein was cannulated with polyethylene tube for administration of supplemental anesthetic and saline (0.9%) infusion. The neck was opened and left thoracotomy was performed to open the thoracic cavity. Anatomy of the left anterior descending coronary artery (LADCA) was examined visually and then ligated 4-5 mm from its origin and the end of this ligature was passed through polyethylene tube to form a snare. The thoracic cavity was covered with saline-soaked gauze to prevent the heart from drying. After completion of the surgical procedure, the heart was returned to its normal position in the thoracic cavity [21, 22].

### 2.8. Estimation of Hemodynamic Variables

The mean arterial pressure (MAP) and heart rate of dogs in all the groups were calculated. The left thoracic cavity was opened by an incision at the fifth intercostal space and the heart was exposed. A sterile metal cannula was introduced in the cavity of the left ventricle from the posterior apical region of the heart for measuring left ventricular dynamics at preset time throughout the surgical procedure [23].

### 2.9. Biochemical Analysis

The blood sampling was performed at different time intervals (0, 12, 24, and 48 hr) during the experimental period. The cardiac biomarkers including creatine kinase-MB (CK-MB), serum glutamic-oxaloacetic transaminase (SGOT), and lactate dehydrogenase (LDH) were analyzed by using “BioMed kits” having patch numbers MBS705376, BGO094144, and LDHK0103016, respectively. All the kits were purchased by “UH Analytics Pakistan.”

### 2.10. Statistical Analysis

The data was statistically analyzed by using two-way ANOVA and Turkey's multiple comparison tests with the help of GraphPad Prism version 7.00, supplied by developer GraphPad software, Inc. [24]. The results have been presented as Mean ± SD.

## 3. Results

### 3.1. Antioxidant Assay

#### 3.1.1. DPPH Free Radical Scavenging Activity

**Figure 1:**
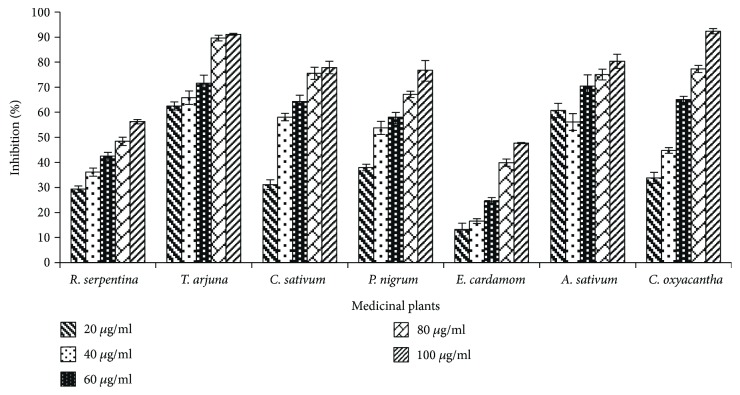
Graphical presentation of antioxidant potential of selected medicinal plants through DPPH radical scavenging activity.

The DPPH free radical scavenging activity (in terms of % age inhibition) of *R. serpentina*, *T. arjuna*, *C. sativum*, *P. nigrum*, *E. cardamom*, *A. sativum*, and *C. oxyacantha* at various concentrations (20, 40, 60, 80, and 100 *μ*g/mL) was examined (Figure 1). The *T. arjuna* and *A. sativum* showed higher antioxidant potential even at least concentration of 20 *μ*g/mL as compared to the same concentrations of other selected plants. On the other hand, the *E. cardamom* presented relatively low antioxidant potential even at its higher concentration of 100 *μ*g/mL. In case of *C. oxyacantha*, the concentration of 20 and 40 *μ*g/mL showed low antioxidative strength but it rapidly increased with further increase in concentration from 60 to 100 *μ*g/mL. All the said medicinal plants depicted the dose-dependent response for free radical scavenging potential, that is, the activity of plant extracts in terms of % age inhibition increased with respect to concentrations (Figure 1). The selected medicinal plants could be beneficial to mankind by virtue of their effective antioxidant activity which may able to impart therapeutic role against various diseases.

### 3.2. DNA Protection Assay

**Figure 2:**
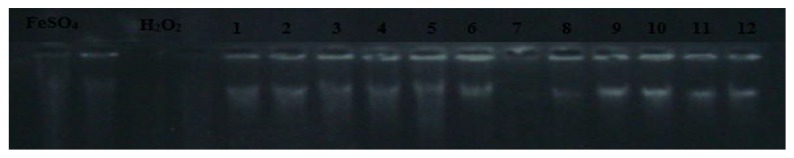
Agarose gel electrophoresis pattern of pBR322 plasmid DNA treated with 30 mM H_2_O_2_ in the presence and absence of different plants extract. [lane 1: pBR322 DNA + 30 mM H_2_O_2_ + P1 (100 *μ*g/mL), lane 2: pBR322 DNA + 30 mM H_2_O_2_ + P1 (500 *μ*/mL), lane 3: pBR322 DNA + 30 mM H_2_O_2_ + P1 (1000 *μ*g/mL), lane 4: pBR322 DNA + 30 mM H_2_O_2_ + P2 (100 *μ*g/mL), lane 5: pBR322 DNA + 30 mM H_2_O_2_ + P2 (500 *μ*g/mL), lane 6: pBR322 DNA + 30 mM H_2_O_2_ + P2 (1000 *μ*g/mL), lane 7: pBR322 DNA + 30 mM H_2_O_2_ + P3 (100 *μ*g/mL), lane 8: pBR322 DNA + 30 mM H_2_O_2_ + P3 (500 *μ*g/mL), lane 9: pBR322 DNA+ 30 mM H_2_O_2_ + P3 (1000 *μ*g/mL), lane 10: pBR322 DNA + 30 mM H_2_O_2_ + P4 (100 *μ*g/mL), lane 11: pBR322 DNA + 30 mM H_2_O_2_ + P4 (500 *μ*g/mL), lane 12: pBR322 DNA + 30 mM H_2_O_2_ + P4 (1000 *μ*g/mL)].

**Figure 3:**
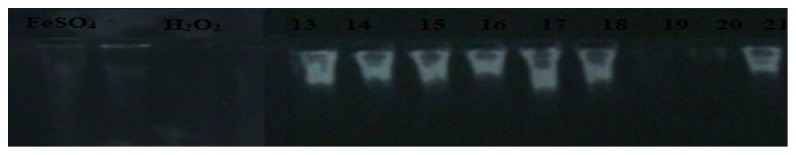
Lane13: pBR322 DNA + 30 mM H_2_O_2_ + P5 (100 *μ*g/mL), lane 14: pBR322 DNA + 30 mM H_2_O_2_ + P5 (500 *μ*g/mL), lane 15: pBR322 DNA + 30 mM H_2_O_2_ + P5 (1000 *μ*g/mL), lane 16: pBR322 DNA + 30 mM H_2_O_2_ + P6 (100 *μ*g/mL), lane 17: pBR322 DNA + 30 mM H_2_O_2_ + P6 (500 *μ*g/mL), lane 18: pBR322 DNA +30 mM H_2_O_2_ + P6 (1000 *μ*g/mL), lane 19: pBR322 DNA + 30 mM H_2_O_2_ + P7 (100 *μ*g/mL), lane 20: pBR322 DNA + 30 mM H_2_O_2_ + P7 (500 *μ*g/mL), lane 21: pBR322 DNA + 30 mM H_2_O_2_ + P7 (1000 *μ*g/mL). P1 = *T. arjuna*; P2 = *C. oxyacantha*; P3 = *P. nigrum*; P4 = *R. serpentina*; P5 = *A. sativum*; P6 = *C. sativum*; P7 = *E. cardamom.*

The effect of varying concentrations of medicinal plants on DNA damage along with positive controls (30% H_2_O_2_, 2 mM FeSO_4_) has been presented in Figures 2 and 3. The free radicals produced in response to O_2_ and FeSO_4_ caused the strand cleavage of pBR322 plasmid DNA and resulted in DNA band streaking (Figure 2). All the plants exhibited protection of plasmid DNA against H_2_O_2_ damage as compared to the plasmid DNA merely treated with H_2_O_2._ The DNA protective potential of all concentrations of said medicinal plants was in concentration-dependent manners which revealed that higher concentrations of extracts are more protective against H_2_O_2_-induced damage. The least concentration (100 *μ*g/mL) of *P. nigrum* showed the band streaking (lane 7 of Figure 2) while the concentration of 500 and 1000 *μ*g/mL of *P. nigrum* exhibited a good protection of pBR322 plasmid DNA as presented in the corresponding lanes 8 and 9. However, in case of *C. oxyacantha*, the concentrations of 100 *μ*g/mL and 500 *μ*g/mL (lanes 4 and 5 of Figure 2) showed noticeable DNA protection. Minor strand breaks were observed with low concentration (100 *μ*g/mL) of *A. sativum* (Figure 2, lane 13), while its higher concentration (500 and 1000 *μ*g/mL) showed promising protection against DNA damage (Figure 2, lanes 14 and 15).

### 3.3. Metabolomic Profiling

Metabolomics approaches using LC-MS-based techniques are a useful technique in evaluating the secondary metabolites of medicinal plants. LC-MS-based metabolomics is a powerful new tool for mechanistic studies of drug metabolism.

### 3.4. *Terminalia Arjuna*

**Figure 4:**
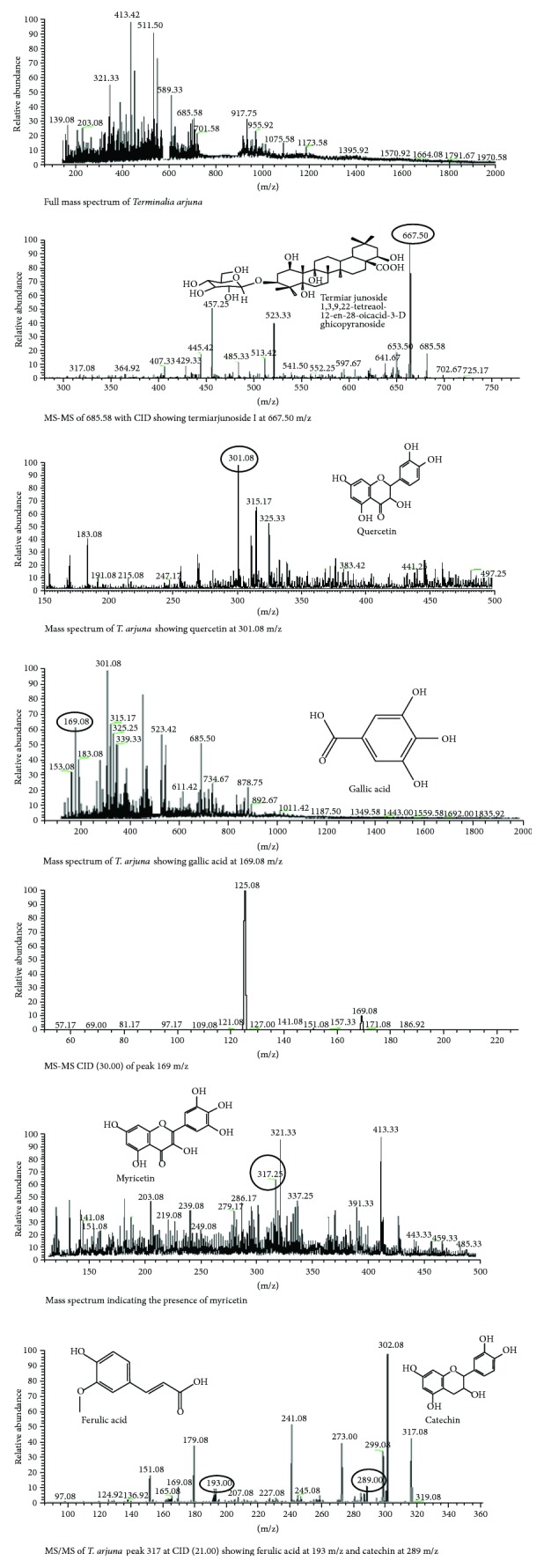
LC-MS analysis of *T. arjuna.*

LC-MS analysis of *T. arjuna* was performed to evaluate the phytoconstituents including phenolics, flavonoids, and alkaloids. The full mass spectrum obtained by LC-MS analysis was presented in Figure 4. The mass spectrum depicted the high peaks at 413.42, 511.50, 321.33, 589.33, and 685.58. The CIDMS-MS-ESI fragments ion of 685.58 peak resulted in three abundant peaks at 667.50, 523.33, and 457.25. The peak of 667.50 indicated the presence of termiarjunoside 1,3,9,22-tetraol-12-en-28-oicacid-3-D-glucopyranoside. The presence of termiarjunoside I from the bark of *T. arjuna* was also reported in a study by Ali et al. 2006. The fast atom bombardment mass spectroscopy (FABMS) of *T. arjuna* also displayed a molecular ion peak at m/z = 666 [M]^+^ indicating the presence of termiarjunoside I, with a molecular formula of C_36_H_58_O_11_, which was also supported by ^13^C and distortionless enhancement by polarization transfer (DEPT) NMR spectra. The mass spectrum revealed the highest peak at 301.08, 317.25, and 169.08 which indicate the presence of quercetin, myricetin, and gallic acid in *T. arjuna*. The presence of gallic acid was further confirmed by MS-MS using CID (30.00). The peak at 125.08 is the consequence of the removal of COO^−^ from gallic acid. The MS-MS of the peak 317.25 by CID (21.00) showed the highest peaks at 302.08, 241.08, and 179.06. However, the peaks at 193 and 289 may indicate the presence of ferulic acid and catechin, respectively.

### 3.5. *Crataegus oxyacantha*

**Figure 5:**
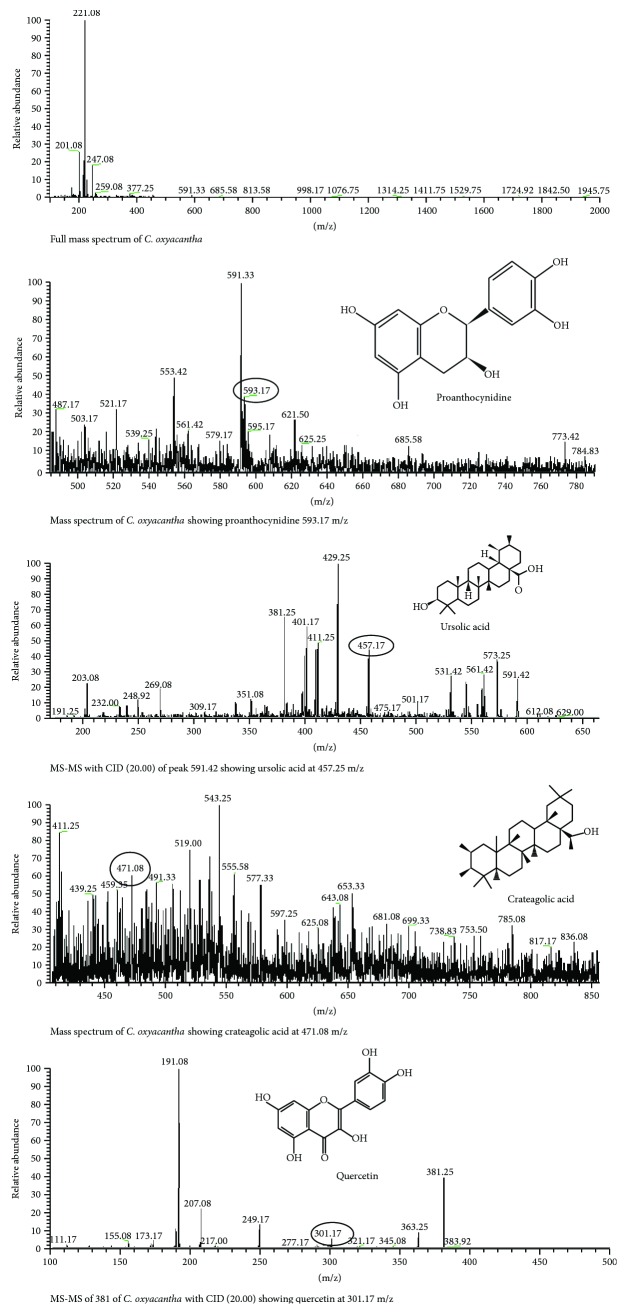
LC-MS analysis of *C. oxyacantha.*

The LC-MS analysis of *C. oxyacantha* was executed to assess the phytoconstituents. The mass spectrum of *C. oxyacantha* showed the peak at 593.17 which indicated the presence of bioactive compounds proanthocynidine with positive mode of ESI (Figure 5). The MS-MS of peak 593 gave the highest peaks at 429.25, 457.17, 411.25, and 401.17, where the peak at 457.17 might indicate the presence of ursolic acid. The LC-MS-ESI also revealed the presence of cratagolic acid at peak of 417 (m/z). The CID MS-MS of the peak 381 of *C. oxyacantha* showed the peak at 301.17 may give the idea of the presence of quercetin.

### 3.6. *Rauwolfia serpentina*

**Figure 6:**
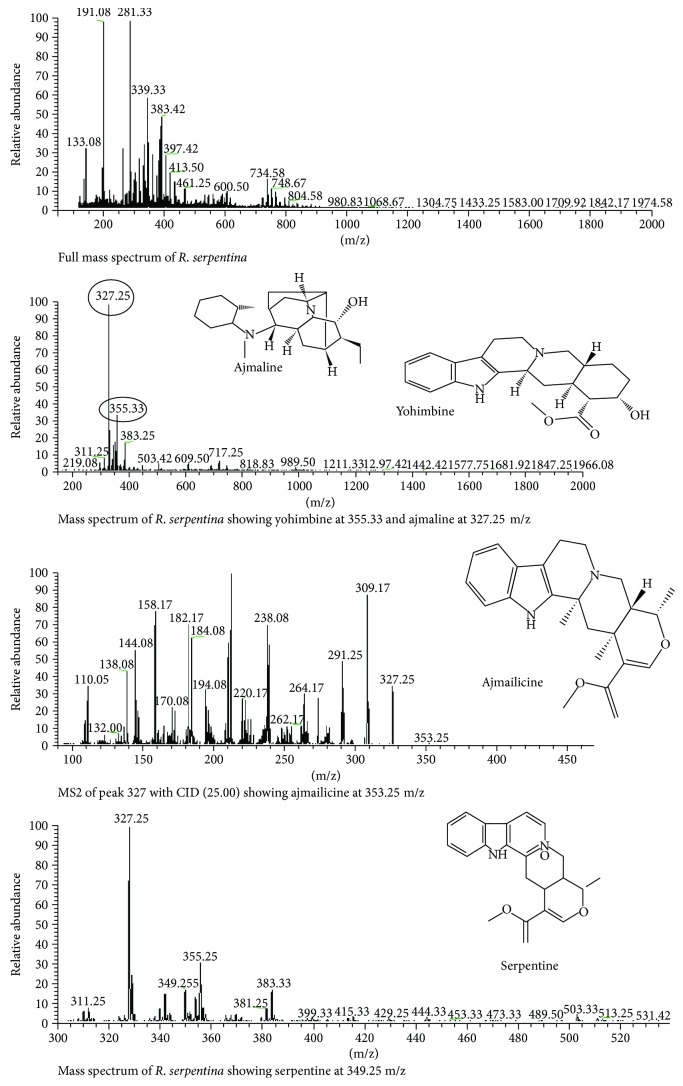
LC-MS analysis of *R. serpentina.*

The LC-MS analysis of roots extract of *R. serpentina* was performed. The full mass spectrums along with the highest peaks at 327.25 and 355.33 indicated the presence of ajmaline and yohimbine, respectively (Figure 6). The MS-MS with CID of 25.00 at peak 327 produced different fragment ion peaks. Among these peaks, the peak at 353.25 m/z may indicate the presence of ajmailacine. The mass spectrum of *R. serpentina* also depicted the presence of serpentine at the peak 349.52.

### 3.7. *Allium sativum*

**Figure 7:**
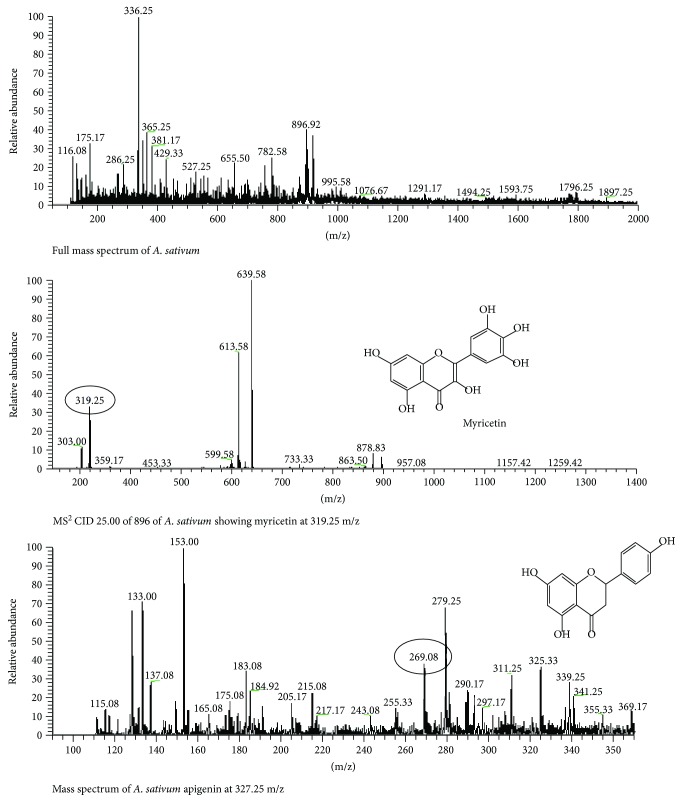
LC-MS analysis of *A. sativum.*

*A. sativum* was subjected to LC-MS analysis to evaluate the presence of phytoconstituents that might be responsible for cardiovascular diseases, dyslipidemia, and hypertension. The LC-MS analysis of *A. sativum* depicted the highest peaks at 896.92, 917.75, and 782.58 (Figure 7). The MS-MS of 896.92 with CID (25.00) gave the peak at 319.25, which indicated the presence of myricetin. The mass spectrum also showed the presence of apigenin at peak 269.08 with negative mode of ESI.

### 3.8. *Coriandrum sativum*

**Figure 8:**
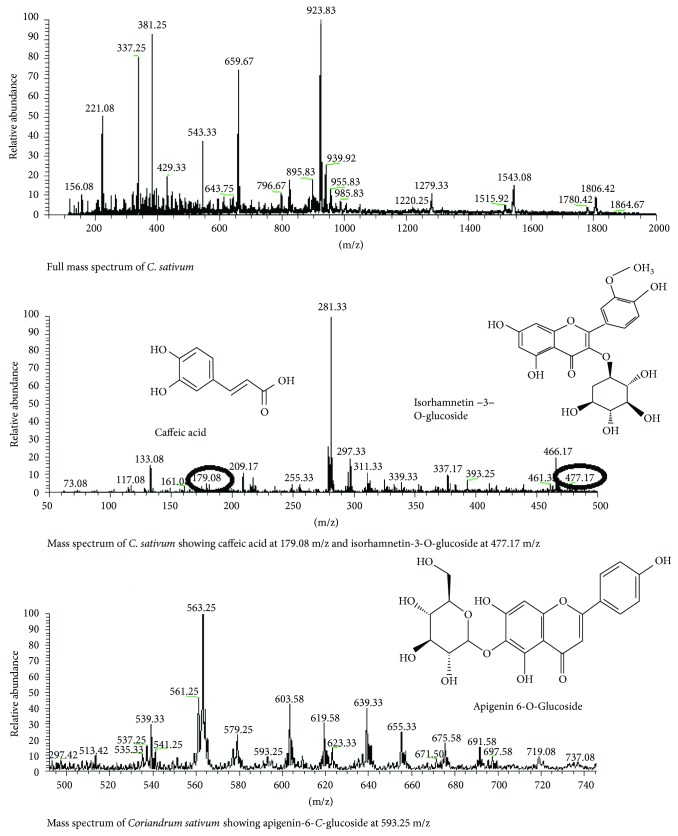
LC-MS analysis of *C. sativum.*

LC-MS analysis of the seed extract of *C. sativum* was performed to evaluate the active phytoconstituents including phenolics, flavonoids, and alkaloids. The full mass spectrum indicated the existence of caffeic acid at peak 179.08 and isorhamnetin-3-O-glucoside at 478.17 m/z. The mass spectrum of *C. sativum* also showed apigenin-6-*C*-glucoside at peak 593.25 m/z with negative mode of electrospray ionization (Figure 8).

### 3.9. *Elettaria cardamom*

**Figure 9:**
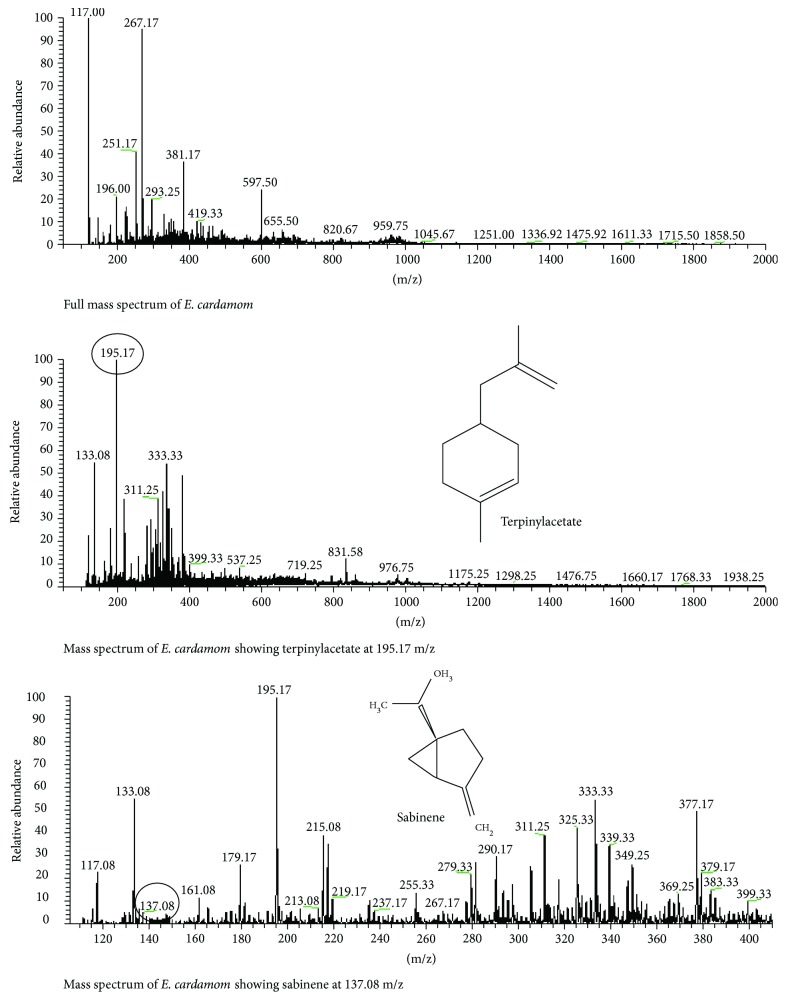
LC-MS analysis of *E. cardamom.*

The mass spectrum obtained by LC-MS analysis of *E. cardamom* represented the high peaks at 195.17133.06 and 333.33. The peak at 195.17 indicated the presence of terpinyl acetate. The mass spectrum also depicted the presence of sebinen at 137.08 peak (Figure 9).

### 3.10. *Piper nigrum*

**Figure 10:**
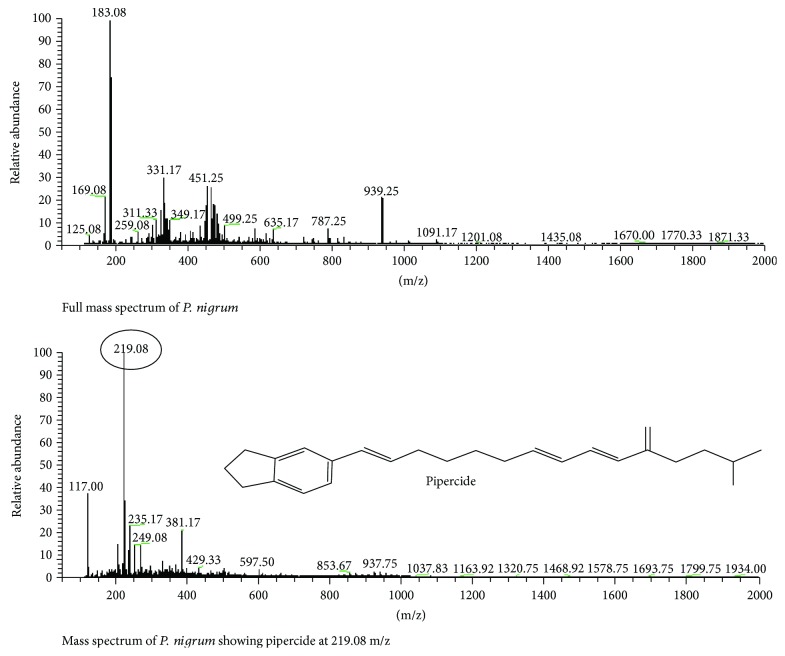
LC-MS analysis of *P. nigrum.*

The methanolic extract of *P. nigrum* is subjected to LC-MS analysis to determine its bioactive compounds that impart crucial role in cardioprotection. The pippercide, an active ingredient of *P. nigrum*, showed its peak at 219.08 (Figure 10).

### 3.11. *In Vivo* Analysis

#### 3.11.1. Effect of Herbal Combinations on Cardiac Markers

To investigate whether the combinations of herbal extracts under investigation would offer any added advantage over individual herbal treatment, the effects of HCs were compared with normal and the surgically induced MI group. The potential of herbal combinations was evaluated by analysing the cardiac markers including CK-MB, SGOT, and LDH.

**Figure 11:**
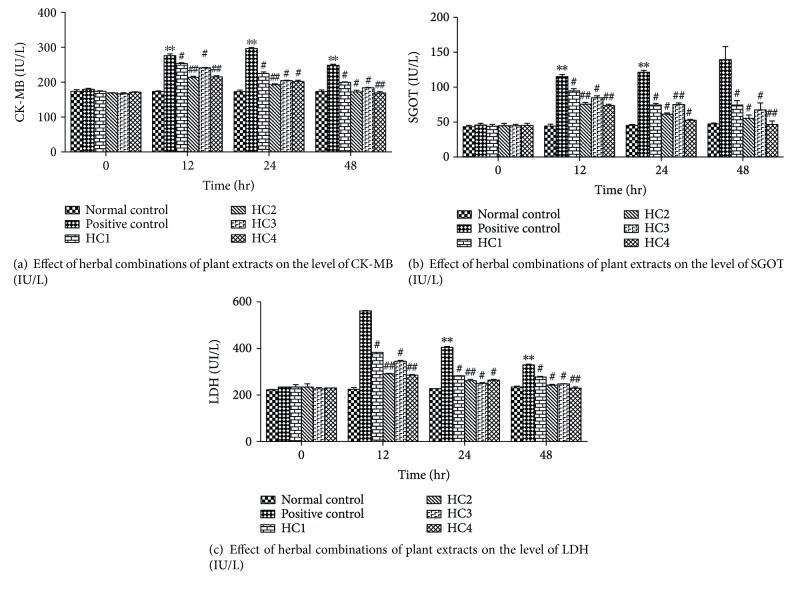
(a–c) Effect of herbal combinations of plant extracts on cardiac markers in the serum of all experimental groups. ∗∗ indicates significance (*p* < 0.0001) compared to the normal control, # indicates significance (*p* < 0.001) compared to the positive control, and ## indicates significance (*p* < 0.0001) compared to the positive control (ANOVA, Turkey's multiple comparison test). Values are presented as the mean ± SEM (*n* = 3).

The effect of different herbal combinations on CK-MB level against surgically induced MI has been presented in Figure 11(a). The normal control group showed the normal CK-MB level (173 ± 3.51 IU/L) throughout the experimental period. There was a considerable increase in the level of CK-MB in the positive control group after 12 hr of left anterior descending coronary artery ligation while the level of enzyme was further raised up to 294.3 ± 1.53 IU/L after 24 hr. The first herbal combination (HC1) did not significantly (*p* > 0.05) restored the CK-MB level after 12 and 24 hr of ligation as compared to the normal control group. In comparison of HC1, the group pretreated with HC2 showed better maintenance of CK-MB level after 12 and 24 hr of ligation. A decrease in CK-MB level was observed in group pretreated with HC4 after 12 hr of ligating left anterior descending coronary artery. After 24 hr of ligation, this group showed considerable decline in the level of CK-MB that was very close to the control group. The prior administration of HC4 depicted the better maintenance of the serum CK-MB as compared to other herbal combinations.

The effect of different herbal combinations on the level of SGOT has been presented in Figure 11(b). In the normal control group, the SGOT level was 43 ± 2 and 46 ± 1.05 IU/L with time intervals of 12 and 24 hr, respectively. The SGOT level was 115 ± 1.527 IU/L and 123 ± 1.154 IU/L after the corresponding time intervals of 12 and 24 hr of LADCA ligation in the positive control group. The HC1 showed the SGOT level with a value of 94 ± 1.53 IU/L after 12 hr and 74 ± 1 IU/L after 24 hr of ligation. The pretreatment of HC2 significantly (*p* > 0.05) maintained at the level of SGOT after 24 hr of ligation in LADCA as compared to the positive control group. There was no considerable variation in the outcomes of HC1 and HC3 preventive treatment. However, the pretreatment of HC4 showed maximum potential against myocardial infarction as it upholds the SGOT level 73 ± 1 IU/L after 12 hr and 53 ± 1.53 IU/L after 24 hr of LADCA ligation.

The preventive treatment of herbal combinations against surgically induced MI on the level of LDH has been presented graphically in Figure 11(c). The serum analysis of the normal control group revealed 223 ± 1.15 to 235 IU/L of LDH from 0 to 48 hr, respectively. The LDH level in the positive control group was considerably higher as compared to the normal control group. The group of dogs pretreated with HC1 showed 382.33 ± 1.53 IU/L of LDH after 12 hr and 283 ± 1.15 IU/L after 24 hr of ligation. In dogs treated with HC2, the LDH level was 291.67 ± 1.15 IU/L and 264 ± 2.08 IU/L at corresponding time intervals of 12 and 24 hr after LADCA ligation. While the pretreatment of HC3 showed 343 ± 1.53 IU/L level of LDH after 12 hr and maintained at the level of 250 ± 1 IU/L after 24 hr of ligation. The preventive treatment of HC4 revealed significant maintenance of LDH level after 12 hr of ligation (Figure 11(c)).

The HC4 showed the prominent cardioprotective potential by maintaining the cardio-specific markers near the normal against surgically induced myocardial infarction after 24 hr of LADCA ligation. Although the precise mechanism of the cardioprotective potential of HCs in surgically induced myocardial injury is not fully understood, it may be attributed to its favorable myocardial adaptogenic properties. Furthermore, this herbal combination might have the potential for the management of patients at risk of myocardial infarction.

### 3.12. Effect of Herbal Combinations on Hemodynamic Variable

#### 3.12.1. The Mean Arterial Pressure

**Figure 12:**
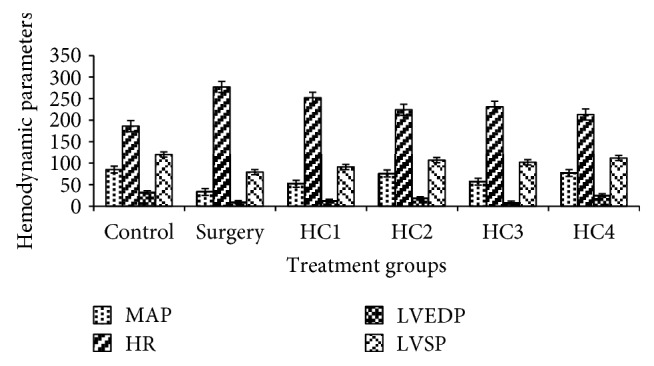
Hemodynamic parameters of various groups treated with different herbal combinations.

Measurement of the hemodynamic variables was also incorporated into the experimental design for better understanding and more precise information of the correlation between biochemical and functional changes in the myocardium subjected to surgically induced damage. The normal control group depicted the 85 ± 6.81 mean arterial pressure (MAP) mmHg while the positive control group showed the decline in MAP (33 ± 4.35 mmHg) after occlusion in LADCA (Figure 12). The pretreatment of HC1 tried to sustain the level of MAP up to 52 ± 5.13 mmHg. However, the group treated with HC2 and HC4 substantially maintained the MAP 76 ± 4.04 mmHg and 77 ± 5.13 mmHg, respectively, as compared to other groups.

Similarly, in the positive control group, there was an abrupt increase in heart rate (HR) beats/min (277 ± 8.02) as compared to the normal control group (186 ± 4.04 beats/min). On the other hand, the pretreatment of surgically induced MI groups with herbal combinations revealed significant (*p* > 0.05) maintenance of HR as compared to the positive control group. Among all the treatment groups, the group pretreated with HC2 and HC4 showed significant (*p* > 0.05) restoration of HR.

#### 3.12.2. Effect of Herbal Combinations on Ventricular Function

A significant decline in left ventricular end-diastolic pressure (LVEDP) (9 ± 3.05) marked the onset of myocardial infarction in surgically induced MI group which remained decreased throughout the experimental period in comparison to the normal control group (32 ± 5.51) (Figure 12). The pretreatment with HC4 and HC2 significantly (*p* > 0.05) maintained the LVEDP level as compared to the surgically induced ischemic group. The HC1 and HC3 also tried to sustain the LVEDP with corresponding values 12 ± 4.04 and 08 ± 1.53.

The positive control group showed the significant decrease in left ventricular systolic pressure (LVSP) as compared to the normal control group. The LADCA ligation resulted in significant cardiac dysfunction evidenced by reduced MAP and increased HR. The left ventricular contractile function was also altered. The pretreatment of HC4 showed the marked restoration as compared to other groups as it maintained the level of LVSP near to the normal control group. It is materialized that the HC4 is more potent in preventing the hemodynamic deteriorations observed in the positive control group.

### 3.13. Histopathological Examination

**Figure 13:**
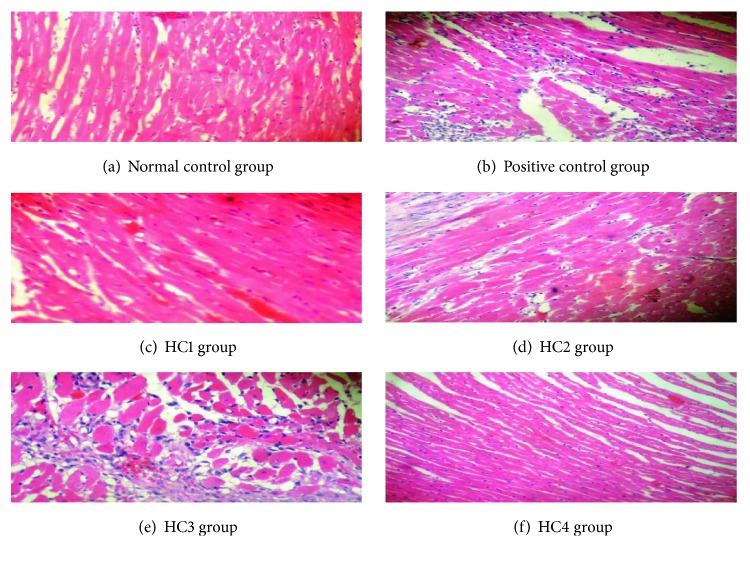
(a–f) The histopathological representation of cardiac tissue of all treatment groups.

The histopathological findings of myocardial tissue in the normal control group illustrated clear integrity of the myocardial cell membrane. The myofibrillar structure was normal with no inflammatory cell infiltration. The nuclei were also normal without any pyknotic changes (Figure 13(a)). The histopathological examination of the surgically induced MI group showed extensive myofibrillar degeneration related to infiltration and disruption of cardiac myofibers. There was marked necrosis in the ventricular region. Pyknotic changes in nuclei were also observed (Figure 13(b)).

The treatment of HC1 prior to ligation showed myofibrilation (Figure 13(c)) while the pretreatment with HC2 demonstrated marked improvement in surgically induced alterations, but there was cellular infiltration at few places. The nuclei were also normal (Figure 13(d)). The group treated with HC3 did not protect the cardiac dysfunctions as compared to the other groups. Myocardial fibrillation as well as some pyknotic changes in the nuclei were also seen in the group treated with HC3 (Figure 13(e)). The histopathological examination of the group treated with HC4 showed that there was no myofibrilation, and the cardiac parenchyma was also normal. This confirmed the potential of herbal combination (HC4) over oxidative stress related to cardiac ailment (Figure 13(f)).

## 4. Discussion

The evidence-based study about metabolomes of medicinal plants is an emerging approach to develop a new group of phytotherapeutics [16]. The therapeutic potential of plant secondary metabolites has augmented an interest in pharmaceutical research for the development of novel therapeutic agents. The antioxidant profiling of the said medicinal plants was explored through DPPH and DNA protection assay. The antioxidative potential of these medicinal plants was found to be dose-dependent. This dose-dependent response of various medicinal plants for antioxidative potential has already been reported by many researchers [18, 25, 26]. The increased antioxidant potential with high dose of medicinal plants may be due to positive correlation with high quantity of powerful chain-breaking antioxidants like phenolics and other phytoconstituents [27]. Different mechanisms like scavenging of free radicals, chelation of metal ions, and inhibition of enzymes may be responsible for good therapeutic antioxidant potential of medicinal plants [28].

In HPLC, the extremely narrow peaks are generated; thus, the high-speed data handling performance demands a blend of MS segment [29]. LC-MS has such features that make it applicable for metabolomic profiling of a wide range of low to high polarity metabolites, including nonvolatile compounds. It also covers a broad range of metabolites, since it operates ionization in negative and positive modes [30]. Hence the LC-MS-based metabolomics is a powerful tool in order to evaluate the important active secondary metabolites which play a vital role to prevent oxidative stress by scavenging free radicals.

The LC-MS analysis of *T. arjuna* revealed the presence of some important phytoconstituents like termiarjunoside I, quercetin, ferulic acid, and gallic acid which were responsible for its antioxidative strength. The HPLC analysis of the *T. arjuna* bark by Jahan et al. [17] also exhibited the existence of polyphenols and phenolic acids including ferulic acid, gallic acid, caffeic acid, and catechin. The fast atom bombardment mass spectroscopy (FABMS) and distortionless enhancement by polarization transfer (DEPT) NMR spectra of *T. arjuna* also displayed a molecular ion peak at m/z = 666 [M]^+^ indicating the presence of termiarjunoside I, with a molecular formula of C_36_H_58_O_11_ (Ali et al. 2006). The quercetin and gallic acids are strong antioxidants which play a crucial role in a number of biological and pharmacological activities and also protect DNA damage [31]. The ferulic acid present in *T. arjuna* is not only a good antioxidant in various biological systems but also has the potential to protect the DNA against H_2_O_2_-induced damage [32].

The metabolomic profiling of *C. oxyacantha* depicted the presence of procynidine, crateagolic acid, ursolic acid, and quercetin. These major phytoconstituents are mainly responsible in curing various diseases like myocardial infarction, coronary heart diseases, hypertension, and diabetes-related complications owing to their antioxidant potential [33]. The presence of ursolic acid in *C. oxyacantha* has also been reported to have angiotensin-converting enzyme-inhibiting and cardioprotective potential (Lacaille et al. 2001). *R. serpentina* has been a popular field of research for decades, and several researchers have explored its excellent phytochemical properties [34, 35]. Various secondary metabolites such as yohimbine, ajmaline, serpentine, and ajmalicine present in the roots of *R. serpentina* contribute for its antioxidant potential [36]. Ajmaline is a sodium channel blocker that illustrated the instant therapeutic potential when given intravenously. It has also been claimed to stimulate respiration and intestinal movements. Serpentine is useful to prevent the oxidative stress-induced DNA damage, hypertension, cardiovascular, and neurological diseases [37]. R. serpentina is a hopeful herbal option in the pharmaceutical world due to the existence of considerable bioactive compounds in the roots [38]. The LC-MS analysis of *A. sativum* indicated the existence of myricetin and apigenin. The myricetin due to its specific chemical structure counteracts oxidative stress generated as a result of reactive oxygen species [39, 40]. The hydroxylated apigenin is found to inhibit tumor cell proliferation and angiogenesis. Caffeic acid is a potent antioxidant and has several therapeutic properties including antioxidants, anti-inflammatory, and anticarcinogenic. It has been reported that caffeic acid inhibits both lipoxygenase activity and suppresses lipid peroxidation thus completely blocks the production of ROS [41]. Cardamom fruit is used against vesicular calculi, dyspepsia, debility, halitosis, and gastrointestinal disorders [42]. Phytochemical investigation of cardamom has revealed highly bioactive components. High-phenolic compounds, in extracts of all plants, could be considered as the key reason behind the antioxidant potential of the said medicinal plants [43, 44].

During *in vivo* trial, the increased cardiac markers in the positive control group are due to the ligation of the coronary artery. The ligation imparts an additional workload on the remaining viable myocytes that may be unbearable, resulting in pathological alterations [11]. Alterations in integrity, fluidity, and permeability of the myocardial membrane due to ligation have been believed to be a reason for the leakage of cardiac markers [21]. The treatment with HCs might salvage viable myocytes, which are at risk of injury, thus preventing cell loss induced by necrosis [45]. The HC4 showed the prominent cardioprotective potential by maintaining the cardio-specific markers near the normal against surgically induced myocardial infarction. The better maintenance of the cardiac markers with HC4 as compared to other herbal combinations might be due to the presence of synergism of some specific phytoconstituents like crateagolic acid, termiarjinoside-I, ajmaline, and serpentine and antioxidants like quercetin, gallic acid, ferulic acid, and myricetin in it. This may render the myocytes less leaky by preventing myocardial membrane destruction [46]. A considerable fall in MAP and increased HR in the surgically induced MI group indicated hemodynamic impairment and ventricular dysfunction due to increased generation of ROS [22]. A fall in MAP due to coronary occlusion is expected to increase HR and myocardial contractility by activating the baroreceptor reflex, which may subsequently result in reflex vasoconstriction and thus worsening the imbalance, between myocardial oxygen demand and supply [47]. The increase in blood flow through the subendocardial region of the left ventricular muscle is the major consequence of the reduction in LVEDP in the surgically induced infarction group [48]. The therapeutic efficacy of HC4 might be due to the improvement in both inotropic and lusitropic function of the heart and considerable maintenance of antioxidant defense capacity of the myocardium [48].

## 5. Conclusion

The HC4 (*T. arjuna*, *R. serpentina*, *E. cardamom*, and *C. oxyacantha*) considerably ameliorated cardiotoxicity by keeping the levels of biochemical parameters near to normal. The antioxidants property and phytoconstituents of medicinal plants present in this herbal combination might be responsible for its cardioprotective potential. On the basis of this evidence-based study, it can be concluded that the HC4 can be safely used as an alternative product for the management of cardiovascular diseases.

## Figures and Tables

**Table 1:** Different herbal combinations given to treatment group.

Groups	*R. serpentina*	*E. cardamom*	*P. nigrum*	*A. sativum*	*T. arjuna*	*C. oxyacantha*	*C. sativum*
*Herbal ratio*							
HC1	1	0.5	1	0.5	—	—	0.5
HC2	1	0.5	1	0.25	—	1	0.5
HC3	1	1	0.5	—	1	—	0.5
HC4	0.5	—	—	0.5	1	0.5	1
